# Age-related remodeling of the sialoglycans dampens murine CD8^+^ T cell function

**DOI:** 10.1126/sciadv.adw6755

**Published:** 2025-09-26

**Authors:** Hanlin Zhang, C. Kimberly Tsui, Jesse Garcia Castillo, Esther Jeong Yoon Kim, Audrey Evangelista, HengChen Liu, Larry K. Joe, Nicholas Twells, Ellen A. Robey, Lara K. Mahal, Michel DuPage, Andrew Dillin

**Affiliations:** ^1^ Division of Immunology and Molecular Medicine, Department of Molecular and Cell Biology, Howard Hughes Medical Institute, University of California, Berkeley, Berkeley, CA 94720, USA.; ^2^ Division of Immunology and Molecular Medicine, Department of Molecular and Cell Biology, University of California, Berkeley, Berkeley, CA 94720, USA.; ^3^ Department of Chemistry, University of Alberta, Edmonton, T6G 2G2, Canada.

## Abstract

Glycans regulate cellular function, yet how aging affects the glycocalyx remains unclear. Here, we investigate changes in immune cell glycocalyx with age and find that α2,6-linked sialic acid, a glycan epitope associated with inhibitory signaling, is down-regulated in T cells from old animals. This reduction is tightly correlated with age-associated accumulation of effector T cells, which have little to no α2,6-linked sialic acid. To understand how α2,6-linked sialic acid affects T cell physiology, we generated a mouse model with T cell–specific deletion of sialyltransferase gene *St6gal1*. The lack of α2,6-linked sialic acid leads to reduced responsiveness in naïve T cells, leading to impaired T cell responses against *Listeria monocytogenes* infection and tumor growth. PD-1 pathway blockade partially restores *St6gal1*-deficient T cells’ ability to control tumor growth. These findings suggest that α2,6-linked sialic acid is critical for maintaining long-term T cell responsiveness, and its loss may contribute to decreased T cell function with age.

## Teaser

Age-related changes in glycocalyx are associated with reduced T cell responsiveness.

## INTRODUCTION

Aging is accompanied by a decline of the immune system, termed immunosenescence (*1*). This decline is linked with increased susceptibility to infections, autoimmune disease, and cancer. T cells are particularly susceptible to age-related dysfunction due to a combination of factors including thymic involution and clonal expansion in the periphery (*2*, *3*). Without high levels of naïve T (T_N_) cell production in the thymus, the total number of T cells and the proportion of T_N_ available for responding to new pathogens decline substantially (*4*). Increased conversion of T_N_ into memory (T_mem_) or effector (T_eff_) cells with age also occurs due to increased homeostatic proliferation and/or chronic antigen exposure (*5*, *6*). During this process, T cells may up-regulate inhibitory receptors such as Programmed death-ligand 1 (PD-1), Lymphocyte activation gene 3 protein (LAG-3), Cytotoxic T-lymphocyte associated protein 4 (CTLA-4), and T cell immunoglobulin- and mucin-domain-containing 3 (TIM-3), dampening T cell response against infections and tumor cells (*7*). Investigating mechanisms of cellular aging from distinct perspectives can help comprehensively understand T cell aging for restoring immune function and controlling age-related diseases.

The surfaces of all cells and organisms are decorated with a diverse repertoire of glycans, collectively known as the glycocalyx (*8*). These glycans play critical roles in mediating both cell-autonomous and nonautonomous signaling events through interaction with membrane-bound and secreted glycan-binding proteins (*9*, *10*). As immune cell development and activation are tightly controlled by extracellular signals, glycosylation can greatly affect immune cell functions. In T cells, glycans regulate thymic development, differentiation, activation, proliferation, and exhaustion (*11*–*16*). Furthermore, specific changes in asparagine (N)–linked glycosylation on T cells have been shown to impair T cell function in aged individuals, in which the degree of N-glycan branching, modifications that negatively affect receptor clustering and activation, becomes up-regulated on T cells of aged female mice and humans (*17*). How different types of glycosylation become altered with age and their functional consequences is starting to be uncovered.

Here, we investigated the changes in glycosylation on multiple immune cell types across age. We identify a previously unreported reduction in α2,6-linked sialic acid, a terminal glycan epitope typically associated with inhibitory signaling (*18*), specifically on aged T cells. We find that this reduction in α2,6-linked sialic acid is closely associated with activated T cells, which account for a larger proportion of total T cells with age. Using a conditional knockout (KO) mouse model that specifically depletes α2,6-linked sialic acid modifications in T cells, we discovered that T cells lacking α2,6-linked sialic acid are less responsive to activating stimuli. These results are in contrast to the acute removal of all sialic acids from T cell surfaces, which leads to more robust T cell activation and rejuvenates PD-1^+^ exhausted T cells (*19*–*22*), underscoring the importance of understanding the functional differences between sialic acid linkages as well as acute versus chronic alterations to T cell glycocalyx. Together, our results highlight the potential link between age-related loss of α2,6-linked sialic acid and T cell functional decline in immunosenescence and uncover the critical role of α2,6-linked sialic acid in maintaining robust and long-lasting CD8^+^ T cell responsiveness.

## RESULTS

### Profiling of glycocalyx changes during immunosenescence

**Fig. 1.**
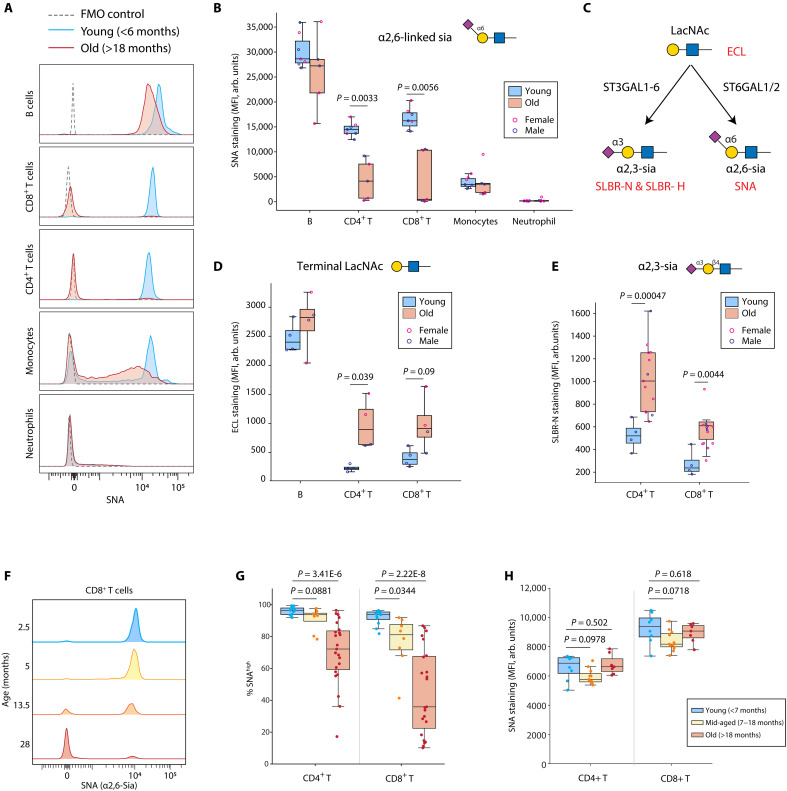
Aging is associated with reduced α2,6-linked sialic acid modification on T cells. (**A** and **B**) Peripheral blood was collected from young (≤6 months, *n* = 7) and old (≥18 months, *n* = 5) mice followed by SNA flow cytometry staining for measuring α2,6-linked sialic acid modifications. B cells (B220^+^), CD4^+^ T cells (CD4^+^), CD8^+^ T cells (CD8^+^), monocytes (Ly6C^+^), and neutrophils (Ly6G^+^) were gated on the basis of their respective surface markers. One representative plot is shown in (A) (young, 1.5 months; and old, 25 months) and summarized in (B). (**C**) Biosynthetic pathways for sialic acid linkages. Lectin specificities are indicated in red. (**D**) Peripheral blood samples from young (*n* = 4) and old (*n* = 4) mice were analyzed by ECL using flow cytometry staining. (**E**) Peripheral blood samples from young (*n* = 4) and old (*n* = 13) mice were analyzed by SLBR-N using flow cytometry staining. (**F** to **H**) Peripheral blood was collected from young (≤6 months, *n* = 12), mid-aged (7 to 17 months, *n* = 8), and old (≥18 months, *n* = 23) mice followed by SNA flow cytometry staining. One representative plot of CD8^+^ T cells staining is shown in (F). The levels of α2,6-linked sialic acid were quantified by percent of SNA^high^ cells in (G) and the mean fluorescence intensity (MFI) of SNA^high^ cells were quantified in (H). All data are represented in box plots showing the quartiles of the data with whiskers showing the rest of the distribution. *P* values were calculated by two-tailed Student’s *t* test.

To understand how aging affects the glycocalyx of immune cells, we measured the levels of different glycan motifs on monocytes, neutrophils, B cells, and T cells in the peripheral blood from young (6 months or younger) and old (18 months or older) mice (see fig. S1A for gating strategy). Specifically, we assayed immature high mannose N-glycans, branched N-glycans, and α2,6-linked sialic acid using lectins *Hippeastrum hybrid* lectin (HHL), *Phaseolus vulgaris*-L (PHA-L), and *Sambucus nigra* (SNA) (*23*), respectively, using flow cytometry (see table S1 for lectin binding specificities). This revealed a slight reduction in high mannose in B cells (fig. S1B; see fig. S8A for staining examples), a reduction in complex branched N-glycans in neutrophils (fig. S1C; see fig. S8B for staining examples), and, most markedly, a loss of α2,6-linked sialic acid in both CD4^+^ and CD8^+^ T cells (Fig. 1, A and B). As α2,6-linked sialic acid motifs are typically the capping sugar residue added to a N-acetyllactosamine (LacNAc) motif (Fig. 1C), we measured the levels of unmasked LacNAc using enhanced chemiluminesence (ECL) and found a corresponding up-regulation in aged T cells (Fig. 1D). In addition, we also find an increase in α2,3-linked sialic acid using Siglec-like binding regions-N and -H (SLBR-N and SLBR-H) [Fig. 1E and fig. S1D; see fig. S8 (C and D) for staining examples] (*24*). However, different from ECL staining results where all SNA^low^ T cells are ECL^+^, the increase in α2,3-linked sialic acid was not found in all SNA^low^ cells (fig. S1, E and F). These results indicate that the exposure of terminal LacNAc may directly result from reduced α2,6-linked sialic acid modification, but age-related up-regulation of α2,3-linked sialic acid modification may have a distinct mechanism.

To gain better time resolution of this sialic acid change, we assayed α2,6-linked sialic acid across age, from 2 to 27 months across immune cell types (fig. S1G). Instead of a gradual linear decrease of SNA staining on T cells, we find that, as mice age, they gain a distinct population of T cells without SNA staining (Fig. 1F). Therefore, we decided to categorize T cells into SNA^high^ or SNA^low^ populations and analyze them separately. We find that, as mice pass the middle age of 18 months, the population of SNA^low^ T cells starts to expand quickly with an increased variability among the aged population (Fig. 1G). This is especially true in CD8^+^ T cells, in which the vast majority of CD8^+^ T cells become SNA^low^ only after 18 months of age. The levels of α2,6-linked sialic acid remain unchanged in the SNA^high^ T cells across age (Fig. 1H), further highlighting that the reduction of α2,6-linked sialic acid in T cells is driven by the loss of this SNA^high^ population.

### Loss of α2,6-linked sialic acid with age is associated with T cell activation

**Fig. 2.**
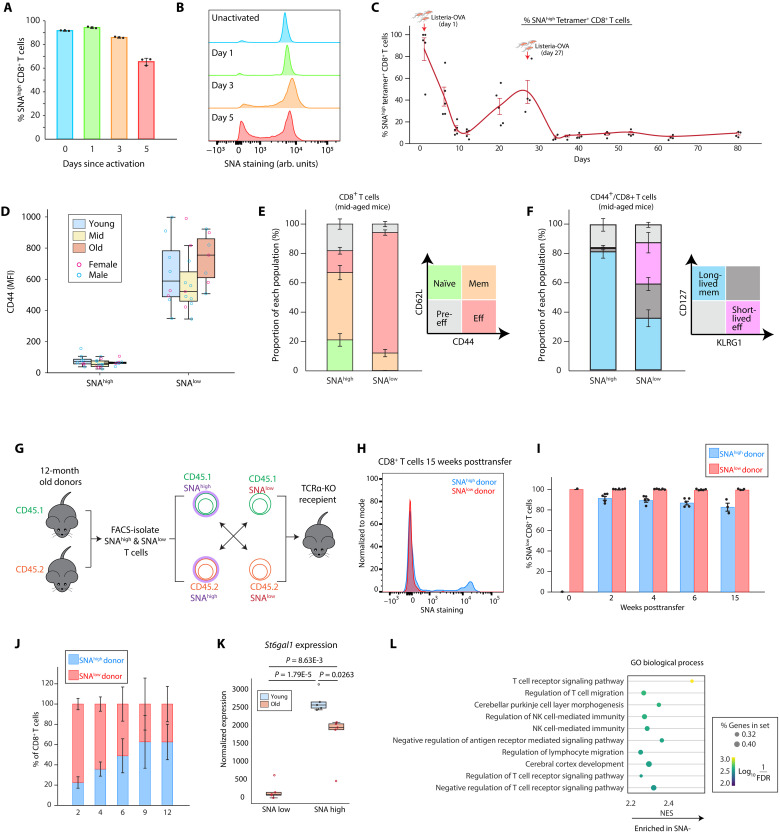
Loss of α2,6-linked sialic acid with age is associated with T cell activation. (**A** and **B**) Splenic T cells from young mice were stimulated with anti-CD3/anti-CD28 for indicated days for SNA staining. Statistical summary (A) and a representative plot (B) is shown. *n* = 3. (**C**) Young WT mice were infected with Listeria-OVA through intravenous injection on day 0 and day 28, respectively. Peripheral blood were collected on indicated days for measuring SNA of OVA tetramer-specific CD8^+^ T cells. *n* = 5. (**D**) Comparison of CD44 levels of SNA^high^ and SNA^low^ CD8^+^ T cells from young (*n* = 8). mid-aged (*n* = 11), or old (*n* = 7) mice. (**E**) SNA^high^ and SNA^low^ CD8^+^ T cells from mid-aged mice were analyzed based on CD44 and CD62L to assess naïve, pre-effector (pre-eff), effector [effector memory (eff)], and memory [central memory (mem)] subpopulations. *n* = 10. (**F**) SNA^high^ and SNA^low^ CD44^+^ CD8^+^ T cells from mid-aged old mice were analyzed based on CD127 and KLRG1 to assess short-lived effector (KLRG1^+^CD127^−^) and long-lived memory (KLRG1^−^CD127^+^) subpopulations. *n* = 10. (**G** to **J**) SNA^high^ and SNA^low^ CD8^+^ T cells were FACS-isolated from CD45.1^+^ or CD45.2^+^ mice respectively, mixed at 1:1 ratio, and adoptively transferred into TCRα-KO recipient mice (G). Peripheral blood were collected at indicated time points for staining. A representative plot is shown in (H) and quantified results are shown in (J). The competitive contribution to the reconstituted population from different donors is quantified in (J). *n* = 5 (**K** and **L**). SNA^high^ and SNA^low^ T cells were sorted from young (only having SNA^low^ cells) and old mice for RNA-seq analyses. The expression of the *St6gal1* gene is shown in (K) and the GO pathway analysis result is shown in (L). NES, normalized enrichment score. *n* = 5. All data represented as means ± SEM. *P* values were calculated by two-tailed Student’s *t* test. NK, natural killer.

Given that T cell activation has been shown to down-regulate α2,6-linked sialic acid (*14*), we hypothesized that the age-related loss of SNA^high^ CD8^+^ T cells is related to increased activation of T cells. We first tested whether activation of young T cells will result in the loss of α2,6-linked sialic acid. In concordance with previous reports, ex vivo activation by anti-CD3/anti-CD28 and in vivo activation by *Listeria monocytogenes* infection both result in the reduction of the proportion of SNA^high^ and a corresponding appearance of SNA^low^ CD8^+^ T cells (Fig. 2, A to C), consistent with changes that we see in aged mice.

To directly test whether the age-related sialic acid changes may result from activation, we costained T cells from young and old mice with a panel of activation markers. We find that, regardless of age, SNA^low^ CD8^+^ T cells are positive for the activation marker CD44 (Fig. 2D and fig. S2A). Moreover, within the CD44^+^ antigen-experienced T cell population in mid-aged mice, SNA^low^ cells are mostly CD44^+^/CD62L^−^ effector T cells, while SNA^high^ cells are more likely to be CD44^+^/CD62L^+^ memory (central memory) T cells (Fig. 2E). To determine whether these SNA^low^ CD8^+^ T cells are long-lived effector memory or short-lived effector cells, we additionally stained CD127 and Killer cell lectin-like receptor subfamily G member 1 (KLRG1) and found that a significant ratio of SNA^low^ CD8^+^ T cells are short-lived effector T cells (CD127^−^/KLRG1^+^), whereas the SNA^high^ CD8^+^ T cells are primarily long-lived memory or memory-precursor cells (CD127^+^/ KLRG1^−^) (Fig. 2F) (*25*).

To more directly evaluate the developmental relationship between SNA^high^ and SNA^low^ T cells, i.e., whether they can transform into each other, we performed a reconstitution experiment by mixing SNA^high^ T cells from CD45.1^+^ mice and SNA^low^ T cells from CD45.2^+^ mice at the 1:1 ratio and adoptively transferred them into T cell receptor alpha (TCRα)–KO recipient mice that lack endogenous T cells (Fig. 2G). We find that, during 15 weeks of reconstitution, SNA^low^ T cells were unable to regain α2,6-linked sialic acid, whereas SNA^high^ T cells can efficiently give rise to SNA^low^ T cells within 2 weeks of reconstitution while retaining a small proportion of SNA^high^ T cells (Fig. 2, H and I). In line with our CD127/KLRG1 staining results, the SNA^low^ CD8^+^ T cells proliferated faster than their SNA^high^ counterpart in recipient mice but were relatively short-lived (Fig. 2J). Therefore, consistent with surface marker costaining results, this functional adoptive transfer experiment further indicates that SNA^high^ T cells are stem cell–like naïve/memory T cells, whereas SNA^low^ T cells are terminally differentiated effector T cells.

As the α2,6-linked sialic acid modification recognized by SNA is mainly generated by the sialyltransferase ST6GAL1, we reanalyzed publicly available single-cell RNA sequencing data of young and old murine immune cells (*26*) to determine whether we can detect any differences in *S6t6gal1* expression in young and old T cells (fig. S2, B and C). In concordance with our SNA staining results (Fig. 1, A and B), *St6gal1* expression is enriched in B cells and, to a lesser degree, T cells, but not in monocytes or neutrophils. However, we were unable to detect sufficient *St6gal1* expression in the single-cell data even in the T_N_ cell population that should all have high levels of α2,6-linked sialic acid, possibly due to the sequencing depth limitation. Therefore, to further understand the differences between the SNA^high^ and SNA^low^ populations of T cells, we performed bulk RNA sequencing to compare these populations of T cells in young and old mice. We find that SNA^high^ T cells have higher expression of *St6gal1* relative to SNA^low^ cells, regardless of age (Fig. 2K), indicating that the loss of α2,6-linked sialic acids is due to transcriptional down-regulation of *St6gal1*. When comparing SNA^high^ and SNA^low^ T cells in aged mice, gene set enrichment analysis further revealed signatures of increased T cell receptor signaling and proliferation in SNA^low^ T cells (Fig. 2L and fig. S2, D and E). Together, these results suggest that the accumulation of activated T cells with age results in the loss of the SNA^high^ T cell population, with reduced *St6gal1* gene expression as the direct cause at the molecular level.

### Loss of α2,6-linked sialic acid leads to altered T cell distribution in the periphery

To study the developmental and functional consequences of the loss of α2,6-linked sialic acid in T cells, we generated a T cell–specific *St6gal1-KO* mice to mimic activation- and age-related down-regulation of *St6gal1* by crossing the *St6gal1^fl/fl^* mice with *CD4-Cre* transgenic mice (*27*). As expected, these *St6gal1-KO* mice have specific depletion of α2,6-linked sialic acid only in their T cells, but not other immune cell types (fig. S3A). They also have increased ECL staining, which binds to the unmasked LacNAc structure that underlies α2,6-linked sialic acids (fig. S3B). α2,3-Linked sialic acid levels are up-regulated in *St6gal1-KO* CD4^+^, but not CD8^+^, T cells, suggesting that there might be a cell-type–specific compensation effect during the sialylation process (fig. S3C).

**Fig. 3.**
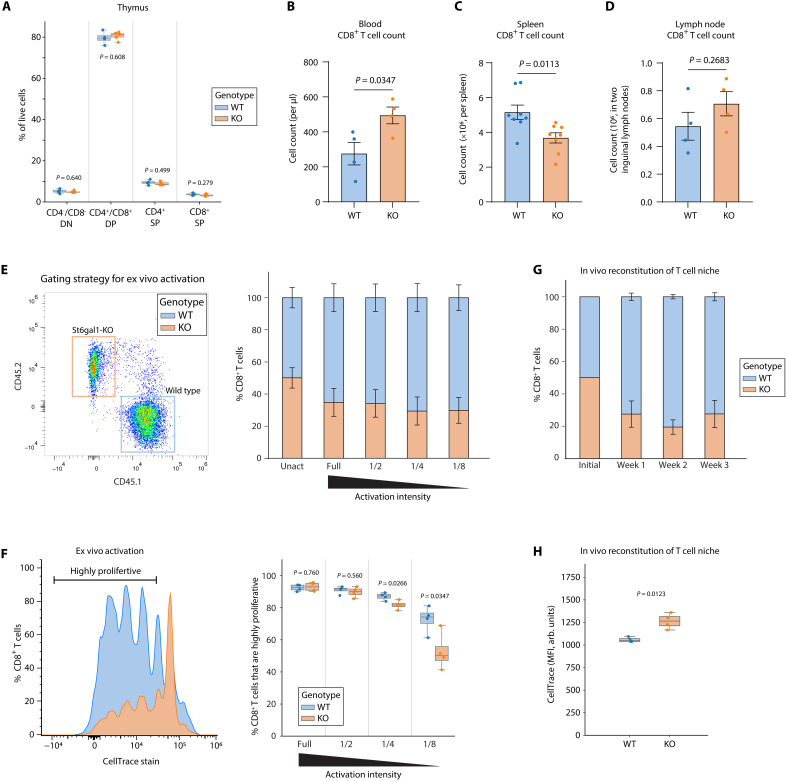
T cells lacking α2,6-linked sialic acids are less proliferative in response to activating stimuli. (**A**) Thymocytes from young CD4-Cre, St6gal1^fl/fl^ mice were analyzed using flow cytometry. *n* = 4 to 5. (**B** to **D**) Flow cytometry quantification of CD8^+^ T cells in the (B) blood, (C) spleen, and (D) inguinal lymph nodes from young CD4-Cre, St6gal1^fl/fl^ mice. *n* = 4 to 8. (**E** and **F**) CD8^+^ T cells from WT (CD45.1^+^) or CD4-Cre, St6gal1^fl/fl^ (CD45.2^+^) mice were mixed at the ratio of 1:1 for ex vivo activation using full dosage of or diluted anti-CD3/anti-CD28 for 3 days (E). Cell proliferation was measured using CellTrace Violet staining with a representative plot shown in [(F), left; 1/8 activation intensity] and quantified in [(F), right]. *n* = 4. Data represented as means ± SEM. (**G** and **H**) CD8^+^ T cells from WT (CD45.1^+^) or CD4-Cre, St6gal1^fl/fl^ (CD45.2^+^) mice were mixed at the ratio of 1:1, stained with CellTrace Violet, and adoptively transferred to TCRα-KO recipient mice. Blood samples were collected at indicated time points to assess reconstitution efficiency (G) and proliferation (H). *n* = 4. Unless otherwise noted, all data are represented in box plots showing the quartiles of the data with whiskers showing the rest of the distribution. *P* values were calculated by two-tailed Student’s *t* test.

Previous findings have reported that T cell–specific depletion of all sialic acids by knocking out the sialic acid synthesis gene *Cmas* led to unaltered thymic T cell development but severe lymphopenia in the periphery with substantially reduced number of mature T cells (*28*). In contrast, whole-body KO of *St6gal1*, which depletes the α2,6-sialic acid modification alone but in a non–tissue-specific manner, led to reduced thymic cellularity and reduced double negative (DN), double positive (DP), and single positive (SP) thymocytes (*29*), indicating that proper sialic acid modification of most likely niche stromal cells is required to maintain thymic T cell survival. Complimentary to these systems, T cell–specific *St6gal1-KO* can be used to properly mimic age-related reduction of α2,6-linked sialic acid on T cells and test how α2,6-sialylation may directly regulate T cell behaviors in an autonomous manner. Deletion of *St6gal1* at the DP stage does not have a significant impact on post–DP stage thymic T cell development, as the proportions of DP and SP CD4^+^ and CD8^+^ thymocytes as well as their subpopulations all remain unchanged (Fig. 3A and fig. S3, D to H). Therefore, consistent with previous reports, our data indicate that sialylation on the T cell surface is dispensable for T cell development and survival during their development in the thymus.

*St6gal1*-KO in T cells leads to an up-regulation of mature T cell numbers in the blood and a corresponding reduction in the spleen, whereas the number of T cells in lymph nodes remains unchanged (Fig. 3, B to D, and fig. S4A). Furthermore, we found no difference in T cell proliferation or T cell death between wild-type (WT) and *St6gal1*-KO mice in the blood (fig. S4, B and C), suggesting that the increased number of T cells in the blood may likely result from reduced splenic migration of T cells rather than increased proliferation or reduced cell death.

To test whether deprivation of α2,6-sialic acid is sufficient to induce T cells activation, we examined the relative proportion of naïve (CD62L^+^CD44^−^), effector (CD62L^−^CD44^+^), and memory (CD62L^+^CD44^+^) CD8^+^ T cells in the blood, spleen, and lymph nodes (fig. S4, D to F). No significant changes were observed among these three populations. However, an emergence of the rare population of CD62L^−^CD44^−^ T cells was observed only in the blood, which may mark a transient status of pre-effector T cells during early T cell activation (fig. S4D) (*30*). Whether the accumulation of this rare population in the blood is due to altered splenic migration or results from other tonic stimulating signals from the microenvironment remains unclear. Moreover, T cell–specific *St6gal1-KO* does not lead to increased overall inflammation as assessed through measuring serum cytokines using an enzyme-linked immunosorbent assay (ELISA) array (fig. S5). Overall, T cell–specific loss of α2,6-linked sialic acid leads to up-regulation of periphery T cells possibly due to impaired splenic migration. These T cells remain phenotypically naïve.

### *St6gal1*-KO T cells are less proliferative in response to activating stimuli

To more directly test the functional consequence of α2,6-linked sialic acid deprivation on CD8^+^ T cells, we measured calcium influx and T cell proliferation in response to TCR stimulation ex vivo. While *St6gal1*-KO and WT T cells show no differences in acute TCR activation sensitivity (fig. S6A), *St6gal1-KO* T cells proliferated less than their WT counterpart under weaker stimuli (Fig. 3, E and F). In line with the reduced ex vivo activation efficiency, *St6gal1-KO* T cells were also less proliferative and less efficient in reconstituting the empty niche in TCRα-KO mice in vivo (Fig. 3, G and H).

**Fig. 4.**
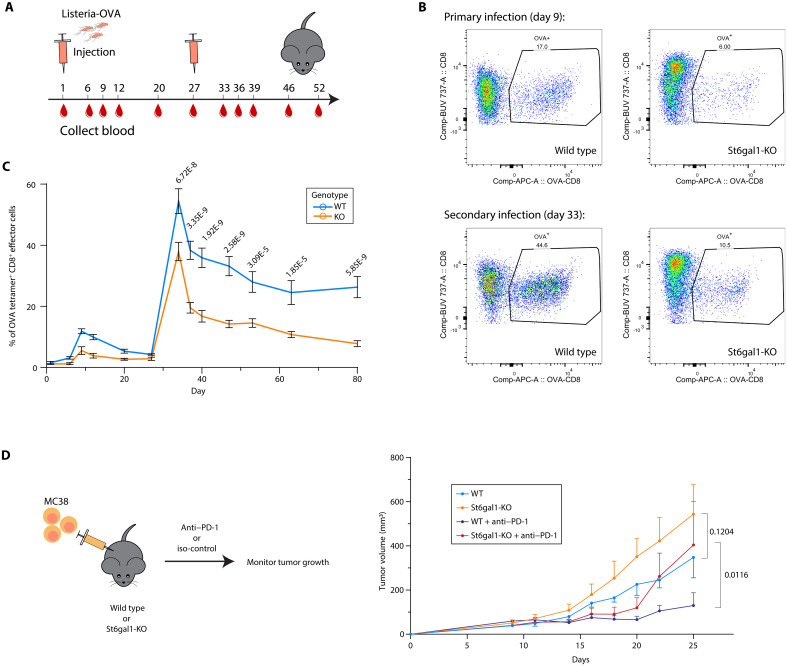
Loss of α2,6-linked sialic acids results in dampened immune response. (**A** to **C**) WT or CD4-Cre, St6gal1^fl/fl^ mice were infected with Listeria-OVA on day 0 and day 28, and blood samples were collected on indicated days (A). OVA antigen-specific CD8^+^ T cells were analyzed using flow cytometry (B) and quantified in (C). *n* = 5 to 6. Data represented as means ± SEM. *P* values are calculated with two-way analysis of variance (ANOVA). (**D**) MC38 tumors were engrafted subcutaneously to WT or CD4-Cre, St6gal1^fl/fl^ mice followed by anti–PD-1 treatment starting from day 11. Tumor volume was measured with time. *n* = 5 to 6. Data represented as means ± SEM. *P* values are calculated with two-way ANOVA with post hoc Dunnett’s test.

Next, we assessed how the specific loss of α2,6-linked sialic acid affects T cell function in various immune activation models in vivo (Fig. 4A). We first used *L. monocytogenes* as an acute infection model, which typically provides a very strong stimulus for T cell activation (*31*). In line with our ex vivo findings, *St6gal1-KO* T cells have a greatly dampened primary response, generating less than half as many antigen-specific CD8^+^ T cells when compared to WT mice (Fig. 4, B and C, and fig. S6B). The corresponding memory response is also weaker (Fig. 4, B and C). Together, these results suggest that the loss of α2,6-linked sialic acid causes T cells to be less responsive and produces a dampened immune response.

Given that *St6gal1-KO* T cells are less responsive to activation stimuli both ex vivo and in vivo, we tested whether the loss of α2,6-linked sialic acid may lead to up-regulation of inhibitory signaling or exhaustion in these T cells. However, circulating *St6gal1-KO* T cells only show minor staining differences in inhibitory receptors LAG-3, CTLA-4, TIM-3, and PD-1 (fig. S6, C to F; see fig. S8E for staining examples). Moreover, these T cells do not express master exhaustion regulators such as thymocyte selection–associated high mobility group box protein (TOX) (fig. S6G) (*32*). These findings indicate that *St6gal1-KO* T cells are not exhausted, and whether their inhibitory signaling is altered to cause functional decline remains to be tested using complementary assays.

As T cell inhibition is particularly relevant to chronic stimulation such as during tumor progression, we tested whether *St6gal1-KO* T cells were also less efficient in controlling tumor growth in an MC38 tumor engraftment model. Consistently, tumors in T cell–specific *St6gal1-KO* mice grow significantly faster than those in WT mice (Fig. 4D). Anti–PD-1 treatment efficiently suppressed tumor growth in these *St6gal1-KO* mice during the first week of treatment (Fig. 4D). This indicates that the loss of α2,6-linked sialic acid on T cells causes reduced responsiveness that can be rescued by checkpoint inhibitor treatment in the short term. However, after 9 days of treatment, tumors in *St6gal1-KO* mice showed increased resistance against anti–PD-1, indicating that multiple inhibitory pathways may be involved in driving the unresponsiveness of T cells lacking α2,6-linked sialic acid.

## DISCUSSION

Dysregulation of T cells is a key contributor to the declining immune system with age, causing older individuals to be more susceptible to a wide array of infectious, autoimmune, and malignant diseases (*2*, *3*). In this study, we investigated how the glycocalyx, which intimately regulates cellular development and function, changes with age on immune cells, particularly CD8^+^ T cells. Using lectin staining on different immune cell types in young and old mice, we identified a marked reduction in α2,6-linked sialic acid modifications specifically in T cells from older animals. Additional experiments reveal that this reduction is linked to age-associated accumulation of T_eff_ cells, which are decorated with little to no α2,6-linked sialic acids. Particularly, these T cells cannot give rise to T cells with high α2,6-linked sialic acids, indicating that this is a lineage-restricted population possibly associated with terminal differentiation. Using a conditional T cell–specific *St6gal1-KO* mouse model, we further discovered that the loss of α2,6-linked sialic acid causes T cells to be less proliferative in response to activating stimuli. Mice without α2,6-linked sialic acid on their T cells are less able to control *Listeria* infection and tumor growth (fig. S7). Last, we show that these T cells can be partially rescued by the blockade of the PD-1 axis, highlighting the importance of α2,6-linked sialic acid in maintaining T cell function.

Previous works have shown that the acute removal of all sialic acid linkages by sialidases on T cells enhances T cell activation (*19*, *21*). However, our data, which strictly focuses on depleting α2,6-linked sialic acid, reveal that this can be detrimental to T cell activity. As sialic acids can play roles in regulating protein secretion and receptor signaling strength, the genetic ablation of *St6gal1* in T cells might alter T cell function through remodeling the T cell proteome and receptor signaling. For example, the function of many inhibitory receptors can be altered by glycosylation (*11*). The removal of α2,6-linked sialic acid might directly change cell surface retention and signaling of these receptors. Future work investigating the differences between acute and chronic depletion of sialic acids on T cells will be important as many sialic acid–modifying therapeutics are being explored for cancer and chimeric antigen receptor (CAR) T cell treatments (*33*–*35*). Furthermore, α2,6-linked sialic acid modification may directly modify inhibitory receptors such as PD-1, LAG-3, TIM-3, and CTLA-4, which are all glycosylated. In particular, CTLA-4 signaling and cell surface retention have been shown to be regulated by N-glycan branching (*36*). How or whether these inhibitory receptors are regulated via α2,6-linked sialic acid modification is unknown and warrants further investigation. Moreover, recent reports have found that human T cell activation is associated with increased α2,8-linked polysialic acid (*37*, *38*). An intriguing possibility for future research is that reduced α2,6-linked sialic acid may lead to compensatory up-regulation of α2,8-linked polysialic acid, which, in turn, suppresses T cell activation. Alternatively, the removal of α2,6-linked sialic acid may expose underlying galactose motifs that can interact with galectins, a family of carbohydrate-binding proteins that have been shown to regulate T cell death and function (*39*–*43*). Further investigation into which galectins specifically bind and alter the T cell response upon depletion of α2,6-linked sialic acid will be important for understanding the underlying mechanism of reduced T cell function.

Age-related reduction of α2,6-linked sialic acid modification was found in both murine CD4^+^ and CD8^+^ T cells. Our current research primarily focuses on CD8^+^ T cells, what about CD4^+^ T cells? It has been reported that antigens modified with sialic acids promote the generation of regulatory T cells and suppress interferon-γ–producing inflammatory CD4^+^ T cells (*44*), raising an intriguing possibility that sialylation may also affect CD4^+^ T cell signaling and functions. It begs further investigation into how sialylation changes during CD4^+^ T cell differentiation and what the acute and chronic impacts of desialylation on different subpopulations of CD4^+^ T cells are. Dendritic cells become more tolerogenic upon taking sialylated antigens with reduced generation of inflammatory cytokines for priming CD4^+^ T cells (*44*). Therefore, sialylation may modify T cell functions via both T cell intrinsic and extrinsic mechanisms. While human and murine T cells do not have exactly the same glycocalyx profiles or glycan remodeling dynamics during ex vivo activation (*45*, *46*), the α2,6-linked sialic acid modification has been shown to also decline in all T cell subsets including CD8^+^, CD4^+^, and γδ T cells upon viral infections in humans (*47*). Further comparison between different T cell subtypes in both human and murine models can generate valuable knowledge about how glycans guide sophisticated T cell dynamics and how evolution adopts appropriate strategies of immune cell glycan remodeling to fit different species with distinct life spans.

Phenotypical biomarkers are useful tools for predicting T cell efficacy in vaccination and immune therapy. A variety of surface markers have been identified as an indicator of different status of T cells, such as CD44, KLRG1, PD-1, and LAG-3 (*48*). Some widely used T cell markers function as receptors for glycans, including CD44 (*49*) and CD62L (*50*). This indicates that glycan-mediated signaling is intimately involved in regulating T cell homeostasis and activation. Our study revealed that α2,6-linked sialic acid depletes when T cells enter a terminally differentiated effector status. Therefore, SNA staining can be used as a binary marker for T cell activation, effector lineage restriction, and dampened response. We also noticed that age-related reduction of the SNA^high^ T cell ratio varies a lot among animals over 18 months old (Fig. 1G). It is possible that the efficacy of their CD8^+^ T cells against infection and tumors also varies in this elder population, which can be predicated by α2,6-linked sialic acid.

In conclusion, our data demonstrate that α2,6-linked sialic acid depletion as indicated by SNA staining can be used as a marker for T cell aging in murine models. This change occurs during CD8^+^ T cell activation and is irreversible under physiological conditions. T cells with low α2,6-linked sialic acid are less proliferative in response to activating stimuli and are less efficient in controlling infection and tumors. Future investigations focused on how α2,6-linked sialic acid regulates human T cell response and aging may provide additional biomarkers and therapeutic strategies for modulating T cell functions.

## MATERIALS AND METHODS

### Animal models

C57BL/6J WT mice (000664, JAX), CD45.1^+^ B6.SJL mice (002014, JAX), *Tcra-KO* mice (002116, JAX), *CD4-Cre* mice (022071, JAX), and *St6gal1-floxed* mice (006901, JAX) were purchased from the Jackson Laboratory. All mice were bred and maintained at the Berkeley Office of Laboratory Animal Care facility until they reach indicated ages for experimental use. Six- to 12-week-old mice were used as young mice for analyses if not specified otherwise. Animal experiments were approved by the local ethical review committee and performed under project licenses AUP-2020-01-12908-1 and AUP-2017-05-9915-2.

### Experimental designs

#### Flow cytometry

Cells were stained with fixable aqua Live/Dead staining (L34966, Thermo Fisher Scientific), FcR block (Thermo Fisher Scientific), and surface marker antibodies in phosphate-buffered saline (PBS) containing 2 mM EDTA at 4°C for 20 min and analyzed with a five-laser LSR Fortessa X-20. Specifically, fluorophore-conjugated lectins, such as SNA (21500045-1, Bioworld; 1:200), ECL (FL-1141-5, Vector Laboratories, 1:200), and SLBR-N and SLBR-H [made in the Mahal laboratory as previously described (*24*)], were stained together with other surface marker antibodies. To stain ovalbumin (OVA) antigen-specific CD8^+^ T cells, the OVA (SIINFEKL)–major histocompatibility complex I tetramer (NIH tetramer SIINFEKL, 1:500) and FcR block were diluted in PBS-EDTA and mixed with cells for staining at room temperature for 1 hour first, followed by staining of other surface markers at 4°C. Forkhead box protein P3 (FOXP3) intracellular staining was performed using the FOXP3 Staining Buffer Set (00-5523-00, Thermo Fisher Scientific) following the manufacturer’s protocol. Acquired data were analyzed using FlowJo 10.8.

#### Adoptive transfer of T cells

SNA^high^ or SNA^low^ CD8^+^ T cells were fluorescence-activated cell sorting (FACS) sorted from mid-aged CD45.1^+^ or CD45.2^+^ donor mice and mixed at the 1:1 ratio. A total of 500,000 donor T cells were adoptively transferred into young *Tcra-KO* recipient mice by intravenous injection. Peripheral blood samples were collected from recipient mice at indicated time points. For transferring WT CD45.1^+^ and *St6gal1-KO* CD45.2^+^ T cells, splenocytes were collected from age-matched young donors by smashing and filtering the spleen tissue through a 70-μm cell strainer and red blood cell lysed. Splenocytes were then analyzed using FACS to measure CD8^+^ T cell ratios and mixed to keep CD8^+^ T cells at the 1:1 ratio. A total of 10,000,000 donor splenocytes were adoptively transferred into young *Tcra-KO* recipient mice followed by peripheral blood sampling at indicated time points.

#### Mouse T cell stimulation

Cell culture plates (96 wells, 3596, Corning) were coated with anti-CD3 antibody of 1 μg/ml (full dose, or diluted to 1/2, 1/4, and 1/8; 16-0032-81, Thermo Fisher Scientific) in PBS at 4°C overnight. On the following day, blood cells were collected from WT CD45.1^+^ and *St6gal1-KO* CD45.2^+^ mice and mixed at the ratio of 1.5:1 (WT: *St6gal1-KO*, so that the ratio of CD8^+^ T cells can be closer to 1:1), followed by red blood cell lysis and CellTrace staining. Mixed blood cells were then resuspended in the T cell culture medium that contains RPMI 1640 (containing Hepes, 22400105; Thermo Fisher Scientific), 10% fetal bovine serum (FBS), penicillin/streptomycin (15-140-122, Thermo Fisher Scientific), GlutaMAX (35050-061, Thermo Fisher Scientific), 50 μM β-mercaptoethanol (M3148, Sigma-Aldrich), and anti-CD28 of 3 μg/ml (full dose, or diluted to 1/2, 1/4, and 1/8; MA110172, Thermo Fisher Scientific) and cultured in the precoated plate for 3 days. About 25 μl of blood was cultured in each well of a 96-well plate. Mixed cells were also analyzed on day 0 to determine the original ratio of CD8^+^ T cells for normalization purpose later.

#### CellTrace staining

To measure cell proliferation, CellTrace Violet (C34557, Thermo Fisher Scientific) was used according to the manufacturer’s protocol. In short, cells were resuspended in 1 ml of CellTrace Violet staining solution (5 μM working concentration, dissolved in PBS) in a 15-ml Falcon tube and incubated in dark at room temperature for 20 min. Then, the staining solution was topped up with RPMI washing media (RPMI 1640 with 10% FBS) and incubated for 5 min to quench the free dye. After centrifugation, cells were washed once more with RPMI washing medium and resuspended in appropriate T cell culture medium that contains indicated amount of anti-CD28.

#### Listeria infection

All strains of *L. monocytogenes* were derived from the WT 10403S strain. All strains were cultured in filter-sterilized nutrient-rich brain heart infusion (BHI) medium (BD Biosciences) containing streptomycin (200 μg/ml; Sigma-Aldrich). Overnight cultures were grown in BHI and streptomycin (200 μg/ml) at 30°C. The following day, bacteria were grown to logarithmic phase by diluting the overnight culture in fresh BHI and streptomycin (200 μg/ml) and culturing at 37°C shaking. Log-phase bacteria were washed and diluted in PBS to infect via the tail vein with 5 × 10^3^ colony-forming units log-phase bacteria. Blood was collected at indicated time points post initial injection for flow analysis.

#### Tumor engraftment

MC38 cell lines were provided by M. DuPage’s laboratory (PMID: 37392735). Cell line were maintained in Dulbecco’s modified Eagle’s medium (11-965-092, Gibco) supplemented with 10% FBS, sodium pyruvate (11-360-070, Gibco), 10 mM Hepes (15-630-080, Gibco), and penicillin-streptomycin (15-140-148, Gibco). Tumor cells were grown at 37°C with 5% CO_2_. For tumor studies, syngeneic C57BL/6J mice were inoculated with 5.0 × 10^5^ MC38 in PBS subcutaneously. Tumor measurements were performed blindly across the entire experiment by a single operator measuring three dimensions of the tumor with calipers three times per week.

#### RNA-seq analysis for SNA^high^ and SNA^low^ T cells

SNA^high^ and SNA^low^ CD8^+^ T cells from mouse thymus of five young and five old mice were isolated by FACS. RNA was purified using an RNeasy Mini Kit (74106, QIAGEN) and stored at −80°C until further processing. RNA-seq libraries were prepared using KAPA mRNA Hyperprep Kit KR1352 and KAPA dual Index Adapters KR1736. NovaSeq PE150 sequencing were performed by MedGenome. RNA-seq analysis was performed by uploading.fastq files to the Galaxy web platform, using the public server at usegalaxy.org, and mouse FASTA reference transcriptome GRCm39 was used for alignment. Tools included Kallisto Quant v0.46.2 + galaxy and DESeq2. Results are available on Dryad (https://doi.org/10.5061/dryad.fqz612k4f). Raw fastq data are available on Sequence Read Archive (SRA) under accession number PRJNA1273472.

#### Reanalysis of single-cell RNA-seq results

Metadata file and Unique molecular identifiers (UMI) counts were obtained directly from Teo *et al*. (*26*) and analyzed using ScanPy (*51*). Only cells expressing 200 to 4000 genes with less than 10% of mitochondrial reads were included in subsequent analysis. Default parameters of ScanPy were used unless otherwise specified. Uniform Manifold Approximation and Projection (UMAP) and Leiden clustering were performed using ScanPy (n_neighbors = 10, n_pcs = 30, and resolution = 0.5). The same cell markers as those by Teo *et al*. (*26*) were used for identifying cell types. They were Cd3e for all T cells, with Cd4 for CD4^+^ T cell and Cd8a for CD8^+^ T cells; Cd79a and Ms4a2 for B cells; Nkg7 for NK cells; Ly6c2 and Cx3cr1 for monocytes or dendritic cells; C1qa, C1qb, and C1qc for macrophage; Fcer1a and Cd200r3 for basophil; and Hba-a1 for erythrocytes. The expression levels of St6gal1 were plotted on the same UMAP.

#### Cytokine array

Serum cytokines were measured using the Proteome Profiler Array (Mouse Cytokine Array Panel A, ARY006, Bio-Techne) according to the manufacturer’s protocol. In short, the array membranes were blocked at room temperature for 1 hour. In the meantime, serum samples collected from mice of indicated genotypes were diluted in the Array Buffer at the ratio of 200 μl:1.3 ml, mixed with 15 μl of reconstituted Mouse Cytokine Array Panel A Detection Antibody Cocktail, and incubated at room temperature for 1 hour. After blocking, the sample/antibody mixture was added to the array membrane and incubated at 4°C overnight on a rocking platform shaker. After washing, the array membrane was incubated with the IRDye 800CW Streptavidin secondary antibody (926-32230, LiCor) and detected using the LiCor Odyssey imaging system. Signals were quantified using Fiji.

#### Indo-1 calcium assay

Twenty million to 30 million freshly prepared splenocytes are resuspended in 1 ml of complete RPMI (cRPMI) with 2 μM Indo-1AM (Invitrogen, I1223). After 30-min incubation in at 37°C protected from light, cells are split between FACS tubes (8 million to 10 million cells per tube), washed with cRPMI, and stained with appropriate surface antibodies followed by viability dye. Washed cells are resuspended in 200 μl of cRPMI and kept on ice until the flow cytometer is ready. Shortly before running samples, each tube of cells is briefly warmed up on a 37°C heat block. After 1 min of acquisition of baseline Indo-1 ratio, appropriate concentration of α-CD3 antibody (Invitrogen, 14-0031-86) is added to tube, briefly vortexed, and another minute of acquisition is followed by addition AffiniPure goat anti-Armenian hamster immunoglobulin G (of 5 μg/ml; Jackson Laboratory, 1277-005-099). Stimulation is observed for 5 min before addition of 1 μM ionomycin for the last minute of acquisition.

### Statistical analysis

All data are presented as means ± SEM or with box and whisker plots showing quartiles and distributions. Experiments are representative of at least two independent experiments unless otherwise indicated. Two groups were compared using the two-tailed Student’s *t* test in Excel. Two-way analysis of variance (ANOVA) with post hoc Dunnett’s test was used for comparing tumor volumes and calculated in Prism. No data were excluded from analyses.

## References

[1] Z. Liu, Q. Liang, Y. Ren, C. Guo, X. Ge, L. Wang, Q. Cheng, P. Luo, Y. Zhang, X. Han, Immunosenescence: Molecular mechanisms and diseases. Signal Transduct. Target. Ther. 8, 200 (2023). 37179335 10.1038/s41392-023-01451-2 PMC10182360

[2] M. Mittelbrunn, G. Kroemer, Hallmarks of T cell aging. Nat. Immunol. 22, 687–698 (2021). 33986548 10.1038/s41590-021-00927-z

[3] J. J. Goronzy, C. M. Weyand, Mechanisms underlying T cell ageing. Nat. Rev. Immunol. 19, 573–583 (2019). 31186548 10.1038/s41577-019-0180-1 PMC7584388

[4] J. E. Mold, P. Réu, A. Olin, S. Bernard, J. Michaëlsson, S. Rane, A. Yates, A. Khosravi, M. Salehpour, G. Possnert, P. Brodin, J. Frisén, Cell generation dynamics underlying naive T-cell homeostasis in adult humans. PLOS Biol. 17, e3000383 (2019). 31661488 10.1371/journal.pbio.3000383 PMC6818757

[5] J. Conway, E. N. De Jong, A. J. White, B. Dugan, N. P. Rees, S. M. Parnell, L. E. Lamberte, A. Sharma-Oates, J. Sullivan, C. Mauro, W. van Schaik, G. Anderson, D. M. E. Bowdish, N. A. Duggal, Age-related loss of intestinal barrier integrity plays an integral role in thymic involution and T cell ageing. Aging Cell 24, e14401 (2024). 39547946 10.1111/acel.14401 PMC11896561

[6] G. Almanzar, S. Schwaiger, B. Jenewein, M. Keller, D. Herndler-Brandstetter, R. Würzner, D. Schönitzer, B. Grubeck-Loebenstein, Long-term cytomegalovirus infection leads to significant changes in the composition of the CD8^+^ T-cell repertoire, which may be the basis for an imbalance in the cytokine production profile in elderly persons. J. Virol. 79, 3675–3683 (2005). 15731261 10.1128/JVI.79.6.3675-3683.2005 PMC1075718

[7] S. Han, P. Georgiev, A. E. Ringel, A. H. Sharpe, M. C. Haigis, Age-associated remodeling of T cell immunity and metabolism. Cell Metab. 35, 36–55 (2023). 36473467 10.1016/j.cmet.2022.11.005 PMC10799654

[8] C. Reily, T. J. Stewart, M. B. Renfrow, J. Novak, Glycosylation in health and disease. Nat. Rev. Nephrol. 15, 346–366 (2019). 30858582 10.1038/s41581-019-0129-4 PMC6590709

[9] A. Varki, Biological roles of glycans. Glycobiology 27, 3–49 (2017). 27558841 10.1093/glycob/cww086 PMC5884436

[10] A. C. Broussard, M. Boyce, Life is sweet: The cell biology of glycoconjugates. Mol. Biol. Cell 30, 525–529 (2019). 30817247 10.1091/mbc.E18-04-0247 PMC6589694

[11] M. S. Pereira, I. Alves, M. Vicente, A. Campar, M. C. Silva, N. A. Padrão, V. Pinto, Â. Fernandes, A. M. Dias, S. S. Pinho, Glycans as key checkpoints of T cell activity and function. Front. Immunol. 9, 2754 (2018). 30538706 10.3389/fimmu.2018.02754 PMC6277680

[12] M. M. Vicente, I. Alves, Â. Fernandes, A. M. Dias, B. Santos-Pereira, E. Pérez-Anton, S. Santos, T. Yang, A. Correia, A. Münster-Kühnel, A. R. M. Almeida, S. Ravens, G. A. Rabinovich, M. Vilanova, A. E. Sousa, S. S. Pinho, Mannosylated glycans impair normal T-cell development by reprogramming commitment and repertoire diversity. Cell. Mol. Immunol. 20, 955–968 (2023). 37344746 10.1038/s41423-023-01052-7 PMC10387478

[13] M. A. Toscano, G. A. Bianco, J. M. Ilarregui, D. O. Croci, J. Correale, J. D. Hernandez, N. W. Zwirner, F. Poirier, E. M. Riley, L. G. Baum, G. A. Rabinovich, Differential glycosylation of T_H_1, T_H_2 and T_H_-17 effector cells selectively regulates susceptibility to cell death. Nat. Immunol. 8, 825–834 (2007). 17589510 10.1038/ni1482

[14] E. M. Comelli, M. Sutton-Smith, Q. Yan, M. Amado, M. Panico, T. Gilmartin, T. Whisenant, C. M. Lanigan, S. R. Head, D. Goldberg, H. R. Morris, A. Dell, J. C. Paulson, Activation of murine CD4^+^ and CD8^+^ T lymphocytes leads to dramatic remodeling of N-linked glycans. J. Immunol. 177, 2431–2440 (2006). 16888005 10.4049/jimmunol.177.4.2431

[15] M. Demetriou, M. Granovsky, S. Quaggin, J. W. Dennis, Negative regulation of T-cell activation and autoimmunity by *Mgat5* N-glycosylation. Nature 409, 733–739 (2001). 11217864 10.1038/35055582

[16] H.-L. Chen, C. F. Li, A. Grigorian, W. Tian, M. Demetriou, T cell receptor signaling co-regulates multiple Golgi genes to enhance N-glycan branching. J. Biol. Chem. 284, 32454–32461 (2009). 19706602 10.1074/jbc.M109.023630 PMC2781660

[17] H. Mkhikian, K. L. Hayama, K. Khachikyan, C. Li, R. W. Zhou, J. Pawling, S. Klaus, P. Q. N. Tran, K. M. Ly, A. D. Gong, H. Saryan, J. L. Hai, D. Grigoryan, P. L. Lee, B. L. Newton, M. Raffatellu, J. W. Dennis, M. Demetriou, Age-associated impairment of T cell immunity is linked to sex-dimorphic elevation of N-glycan branching. Nat. Aging. 2, 231–242 (2022). 35528547 10.1038/s43587-022-00187-y PMC9075523

[18] S. Duan, J. C. Paulson, Siglecs as immune cell checkpoints in disease. Annu. Rev. Immunol. 38, 365–395 (2020). 31986070 10.1146/annurev-immunol-102419-035900

[19] L. J. Edgar, A. J. Thompson, V. F. Vartabedian, C. Kikuchi, J. L. Woehl, J. R. Teijaro, J. C. Paulson, Sialic acid ligands of CD28 suppress costimulation of T cells. ACS Cent. Sci. 7, 1508–1515 (2021). 34584952 10.1021/acscentsci.1c00525 PMC8461770

[20] E. U. Bagriaçik, K. S. Miller, Cell surface sialic acid and the regulation of immune cell interactions: The neuraminidase effect reconsidered. Glycobiology 9, 267–275 (1999). 10024664 10.1093/glycob/9.3.267

[21] A. A. Sadighi Akha, S. B. Berger, R. A. Miller, Enhancement of CD8 T-cell function through modifying surface glycoproteins in young and old mice. Immunology 119, 187–194 (2006). 16805788 10.1111/j.1365-2567.2006.02420.x PMC1782347

[22] S. Sasawatari, Y. Okamoto, A. Kumanogoh, T. Toyofuku, Blockade of *N*-glycosylation promotes antitumor immune response of T cells. J. Immunol. 204, 1373–1385 (2020). 31969386 10.4049/jimmunol.1900937

[23] D. Bojar, L. Meche, G. Meng, W. Eng, D. F. Smith, R. D. Cummings, L. K. Mahal, A useful guide to lectin binding: Machine-learning directed annotation of 57 unique lectin specificities. ACS Chem. Biol. 17, 2993–3012 (2022). 35084820 10.1021/acschembio.1c00689 PMC9679999

[24] B. A. Bensing, Q. Li, D. Park, C. B. Lebrilla, P. M. Sullam, Streptococcal Siglec-like adhesins recognize different subsets of human plasma glycoproteins: Implications for infective endocarditis. Glycobiology 28, 601–611 (2018). 29796594 10.1093/glycob/cwy052 PMC6054165

[25] N. S. Joshi, W. Cui, A. Chandele, H. K. Lee, D. R. Urso, J. Hagman, L. Gapin, S. M. Kaech, Inflammation directs memory precursor and short-lived effector CD8^+^ T cell fates via the graded expression of T-bet transcription factor. Immunity 27, 281–295 (2007). 17723218 10.1016/j.immuni.2007.07.010 PMC2034442

[26] Y. V. Teo, S. J. Hinthorn, A. E. Webb, N. Neretti, Single-cell transcriptomics of peripheral blood in the aging mouse. Aging (Albany NY) 15, 6–20 (2023). 36622281 10.18632/aging.204471 PMC9876630

[27] T. Hennet, D. Chui, J. C. Paulson, J. D. Marth, Immune regulation by the ST6Gal sialyltransferase. Proc. Natl. Acad. Sci. U.S.A. 95, 4504–4509 (1998). 9539767 10.1073/pnas.95.8.4504 PMC22519

[28] M. Schmidt, A. T. Linder, M. Korn, N. Schellenberg, S. J. Meyer, F. Nimmerjahn, A. Werner, M. Abeln, R. Gerardy-Schahn, A. K. Münster-Kühnel, L. Nitschke, Sialic acids on T cells are crucial for their maintenance and survival. Front. Immunol. 15, 1359494 (2024). 38947328 10.3389/fimmu.2024.1359494 PMC11211268

[29] J. H. Marino, C. Tan, B. Davis, E.-S. Han, M. Hickey, R. Naukam, A. Taylor, K. S. Miller, C. J. Van De Wiele, T. K. Teague, Disruption of thymopoiesis in ST6Gal I-deficient mice. Glycobiology 18, 719–726 (2008). 18535087 10.1093/glycob/cwn051 PMC2733770

[30] Y. Nakajima, K. Chamoto, T. Oura, T. Honjo, Critical role of the CD44^low^CD62L^low^ CD8^+^ T cell subset in restoring antitumor immunity in aged mice. Proc. Natl. Acad. Sci. U.S.A. 118, e2103730118 (2021). 34088845 10.1073/pnas.2103730118 PMC8201912

[31] S. I. Mannering, J. Zhong, C. Cheers, T-cell activation, proliferation and apoptosis in primary *Listeria monocytogenes* infection. Immunology 106, 87–95 (2002). 11972636 10.1046/j.1365-2567.2002.01408.x PMC1782690

[32] O. Khan, J. R. Giles, S. McDonald, S. Manne, S. F. Ngiow, K. P. Patel, M. T. Werner, A. C. Huang, K. A. Alexander, J. E. Wu, J. Attanasio, P. Yan, S. M. George, B. Bengsch, R. P. Staupe, G. Donahue, W. Xu, R. K. Amaravadi, X. Xu, G. C. Karakousis, T. C. Mitchell, L. M. Schuchter, J. Kaye, S. L. Berger, E. J. Wherry, TOX transcriptionally and epigenetically programs CD8^+^ T cell exhaustion. Nature 571, 211–218 (2019). 31207603 10.1038/s41586-019-1325-x PMC6713202

[33] J. S. Durgin, R. Thokala, L. Johnson, E. Song, J. Leferovich, V. Bhoj, S. Ghassemi, M. Milone, Z. Binder, D. M. O’Rourke, R. S. O’Connor, Enhancing CAR T function with the engineered secretion of C. perfringens neuraminidase. Mol. Ther. 30, 1201–1214 (2022). 34813961 10.1016/j.ymthe.2021.11.014 PMC8899523

[34] C. Büll, T. J. Boltje, N. Balneger, S. M. Weischer, M. Wassink, J. J. van Gemst, V. R. Bloemendal, L. Boon, J. van der Vlag, T. Heise, M. H. den Brok, G. J. Adema, Sialic acid blockade suppresses tumor growth by enhancing T-cell-mediated tumor immunity. Cancer Res. 78, 3574–3588 (2018). 29703719 10.1158/0008-5472.CAN-17-3376

[35] N. Rodrigues Mantuano, M. Natoli, A. Zippelius, H. Läubli, Tumor-associated carbohydrates and immunomodulatory lectins as targets for cancer immunotherapy. J. Immunother. Cancer. 8, e001222 (2020). 33020245 10.1136/jitc-2020-001222 PMC7537339

[36] K. S. Lau, E. A. Partridge, A. Grigorian, C. I. Silvescu, V. N. Reinhold, M. Demetriou, J. W. Dennis, Complex N-glycan number and degree of branching cooperate to regulate cell proliferation and differentiation. Cell 129, 123–134 (2007). 17418791 10.1016/j.cell.2007.01.049

[37] S. Makhsous, C. Hunter, C. van Eeden, M. Osman, L. Willis, Polysialylation is a general feature of immune activation. bioRxiv 632476 [Preprint] (2025). 10.1101/2025.01.10.632476.

[38] O. Drummond-Guy, J. Daly, A. Wu, N. Stewart, K. Milne, C. Duff, B. H. Nelson, K. C. Williams, S. Wisnovsky, Polysialic acid is upregulated on activated immune cells and negatively regulates anticancer immune activity. Front. Oncol. 15, 1520948 (2025). 40182033 10.3389/fonc.2025.1520948 PMC11965634

[39] F. Cedeno-Laurent, C. J. Dimitroff, Galectin-1 research in T cell immunity: Past, present and future. Clin. Immunol. 142, 107–116 (2012). 22019770 10.1016/j.clim.2011.09.011 PMC3266984

[40] R. C. Gilson, S. D. Gunasinghe, L. Johannes, K. Gaus, Galectin-3 modulation of T-cell activation: Mechanisms of membrane remodelling. Prog. Lipid Res. 76, 101010 (2019). 31682868 10.1016/j.plipres.2019.101010

[41] H.-Y. Chen, A. Fermin, S. Vardhana, I.-C. Weng, K. F. R. Lo, E.-Y. Chang, E. Maverakis, R.-Y. Yang, D. K. Hsu, M. L. Dustin, F.-T. Liu, Galectin-3 negatively regulates TCR-mediated CD4^+^ T-cell activation at the immunological synapse. Proc. Natl. Acad. Sci. U.S.A. 106, 14496–14501 (2009). 19706535 10.1073/pnas.0903497106 PMC2732795

[42] G. Wu, W. Deng, H.-Y. Chen, H.-J. Cho, J. Kim, Galectin 7 leads to a relative reduction in CD4+ T cells, mediated by PD-1. Sci. Rep. 14, 6625 (2024). 38503797 10.1038/s41598-024-57162-3 PMC10951237

[43] N.-W. Kam, C. Y. Lau, J. Y. H. Lau, X. Dai, Y. Liang, S. P. H. Lai, M. K. Y. Chung, V. Z. Yu, W. Qiu, M. Yang, C. Smith, R. Khanna, K. M. Ng, W. Dai, C. M. Che, V. H.-F. Lee, D. L. W. Kwong, Cell-associated galectin 9 interacts with cytotoxic T cells confers resistance to tumor killing in nasopharyngeal carcinoma through autophagy activation. Cell. Mol. Immunol. 22, 260–281 (2025). 39910335 10.1038/s41423-024-01253-8 PMC11868493

[44] M. Perdicchio, J. M. Ilarregui, M. I. Verstege, L. A. M. Cornelissen, S. T. T. Schetters, S. Engels, M. Ambrosini, H. Kalay, H. Veninga, J. M. M. den Haan, L. A. van Berkel, J. N. Samsom, P. R. Crocker, T. Sparwasser, L. Berod, J. J. Garcia-Vallejo, Y. van Kooyk, W. W. J. Unger, Sialic acid-modified antigens impose tolerance via inhibition of T-cell proliferation and de novo induction of regulatory T cells. Proc. Natl. Acad. Sci. U.S.A. 113, 3329–3334 (2016). 26941238 10.1073/pnas.1507706113 PMC4812702

[45] F. N. Izzati, H. Choksi, P. Giuliana, D. Abd-Rabbo, H. Elsaesser, A. Blundell, V. Affe, V. Kannen, Z. Jame-Chenarboo, E. Schmidt, M. Kuypers, D. B. Avila, E. S. Y. Chiu, D. Badmaev, H. Cui, J. Matthews, T. Mallevaey, M. S. Macauley, D. G. Brooks, L. J. Edgar, A unified atlas of T cell glycophysiology. bioRxiv 609521 [Preprint] (2024). 10.1101/2024.08.24.609521.

[46] D. Sierra-Ulloa, J. Fernández, M. Cacelín, G. A. González-Aguilar, R. Saavedra, E. P. Tenorio, α2,6 sialylation distinguishes a novel active state in CD4^+^ and CD8^+^ cells during acute *Toxoplasma gondii* infection. Front. Immunol. 15, 1429302 (2024). 39253089 10.3389/fimmu.2024.1429302 PMC11381403

[47] I. Alves, M. M. Vicente, J. Gaifem, Â. Fernandes, A. M. Dias, C. S. Rodrigues, J. C. Oliveira, N. Seixas, L. Malheiro, M. A. Abreu, R. Sarmento E Castro, S. S. Pinho, SARS-CoV-2 infection drives a glycan switch of peripheral T cells at diagnosis. J. Immunol. 207, 1591–1598 (2021). 34417260 10.4049/jimmunol.2100131

[48] J. S. Dolina, N. Van Braeckel-Budimir, G. D. Thomas, S. Salek-Ardakani, CD8^+^ T cell exhaustion in cancer. Front. Immunol. 12, 715234 (2021). 34354714 10.3389/fimmu.2021.715234 PMC8330547

[49] A. Aruffo, I. Stamenkovic, M. Melnick, C. B. Underhill, B. Seed, CD44 is the principal cell surface receptor for hyaluronate. Cell 61, 1303–1313 (1990). 1694723 10.1016/0092-8674(90)90694-a

[50] A. Ivetic, H. L. Hoskins Green, S. J. Hart, L-selectin: A major regulator of leukocyte adhesion, migration and signaling. Front. Immunol. 10, 1068 (2019). 31139190 10.3389/fimmu.2019.01068 PMC6527602

[51] F. A. Wolf, P. Angerer, F. J. Theis, SCANPY: Large-scale single-cell gene expression data analysis. Genome Biol. 19, 15 (2018). 29409532 10.1186/s13059-017-1382-0 PMC5802054

