# Supplementary material for: Age-related remodeling of the sialoglycans dampens murine CD8+ T cell function
Source: Sci Adv. 2025 Sep 26;11(39):eadw6755. doi: 10.1126/sciadv.adw6755 (PMC12467053; doi:10.1126/sciadv.adw6755)
Supplement: Supplementary file 1 — Figs. S1 to S8 Table S1 [file sciadv.adw6755_sm.pdf]

Science Advances
AAAS

Supplementary Materials for

Age-related remodeling of the sialoglycans dampens murine CD8$^+$ T cell function

Hanlin Zhang *et al.*

Corresponding author: Andrew Dillin, dillin@berkeley.edu

Sci. Adv. **11**, eadw6755 (2025)
DOI: 10.1126/sciadv.adw6755

This PDF file includes:

Figs. S1 to S8
Table S1

Supplementary Figures

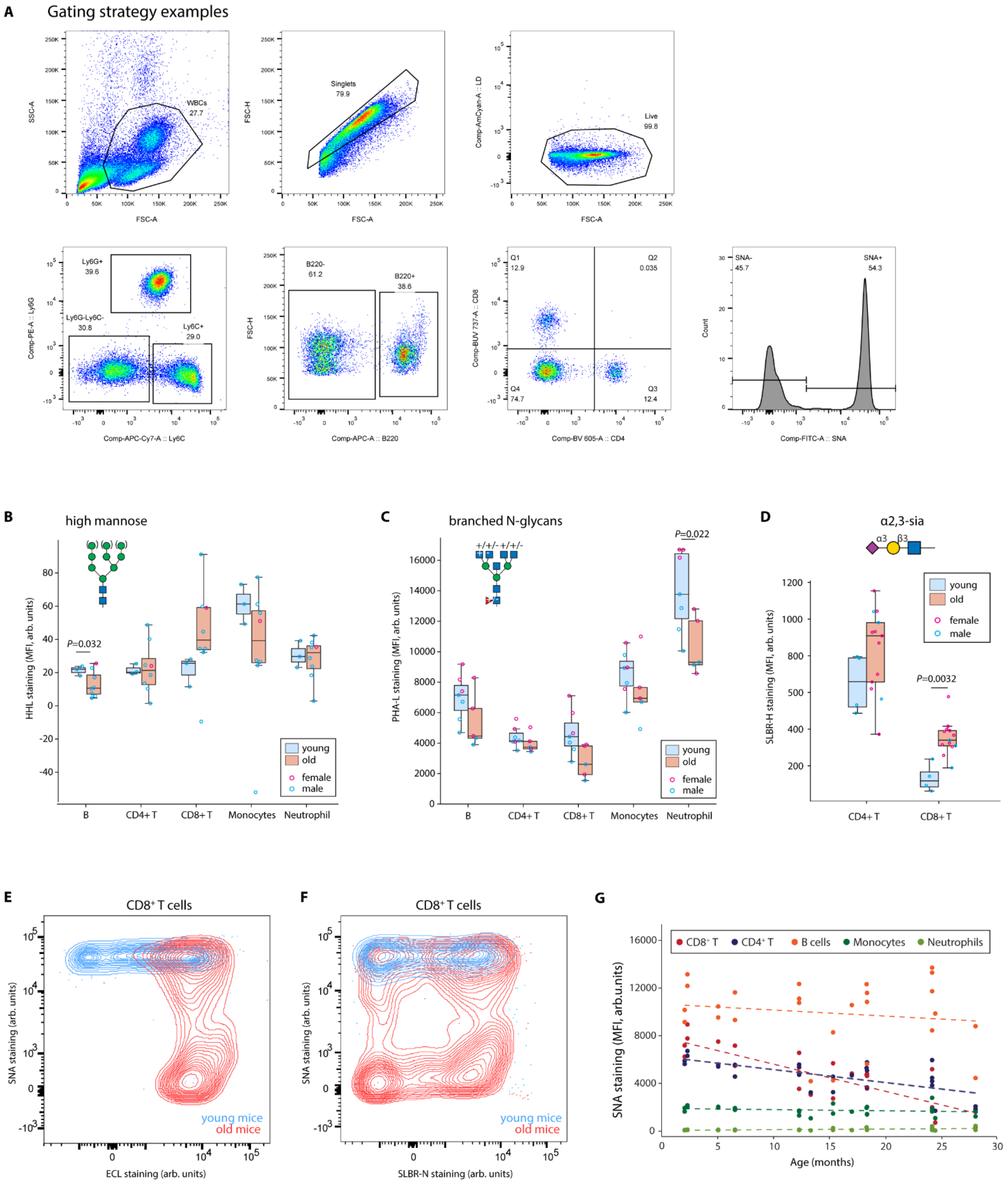

Figure S1. Glycan profiling of immune cells during aging.

(A) Representative flow cytometry graphs of gating strategy for different immune cell types.

(B – D) Peripheral blood was collected from young (< 7 months) and old (>18 months) mice followed by HHL (B), PHA-L (C), and SLBR-H (D) flow cytometry staining for measuring high mannose, branched N-glycans, and α2,3-linked sialic acid modification respectively. n =3-8.

(E -F) A representative plot of SNA/SLBR-N (E) or SNA/ECL (F) co-staining of $CD8^+$ T cells from young and old mice.

(G) Peripheral blood samples from mice of indicated ages were analyzed for α2,6-linked sialic acid modification by SNA flow cytometry staining. n = 7-11.

Data are represented in box plots showing the quartiles of the data with whiskers showing the rest of the distribution. *P* values were calculated by two-tailed student's t-test.

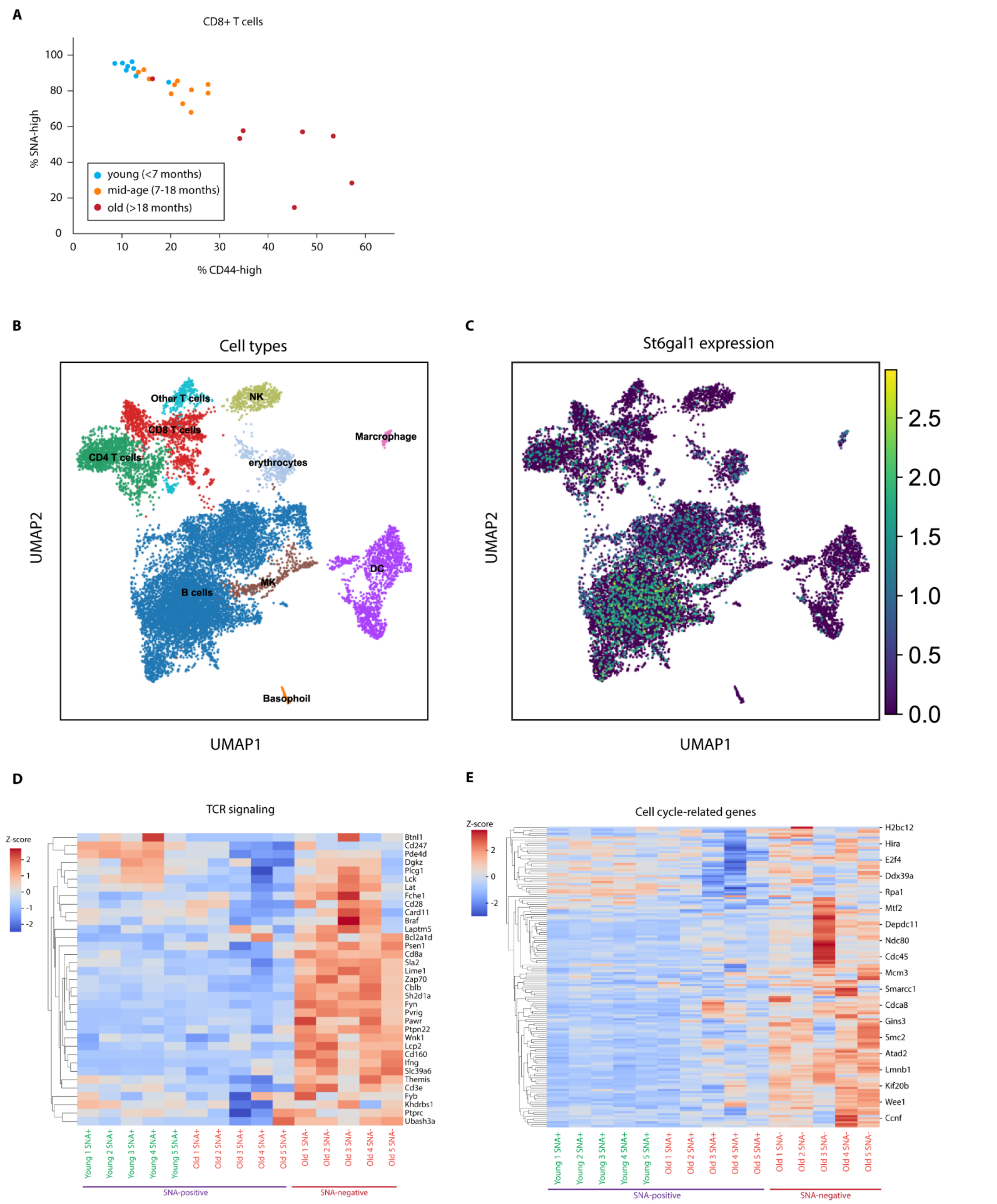

Figure S2. Subpopulation analysis of young and old immune cells using single cell RNA-seq and bulk RNA-seq.

(A) Comparison of percentage of CD8+ T cells that are SNA^{high} and $CD44^{high}$ from young (n=8), mid-age(n=11) or old (n=7) mice.

(B) Single-cell RNA-seq data of immune cells from young and old mice blood were analyzed using UMAP.

(C) The expression of *St6gal1* was highlighted in the UAMP.

(D – E) SNA^+ and SNA^- T cells (including both CD8+ and CD4+) from young and old mice were FACS-sorted for bulk RNA-seq analysis. Most significantly changed pathways are shown, such as TCR signaling (D) and cell cycle-related genes (E). n =5.

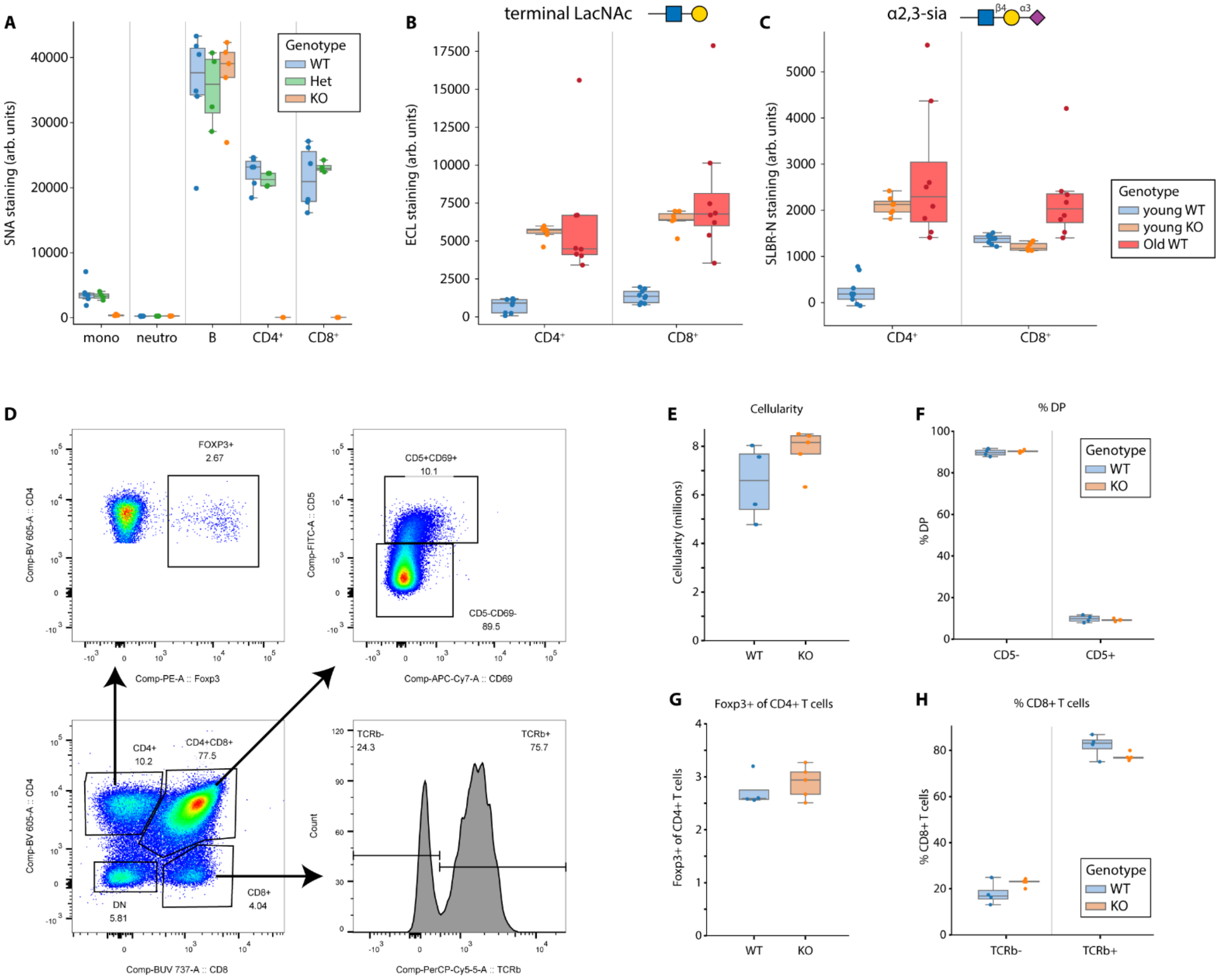

Figure S3. T-cell specific knockout of *St6gal1* does not significantly affect T cell development.

(A) Peripheral blood samples were collected from wildtype (WT, littermate control), *CD4-Cre, St6gal1-flox/wt* (Het), or *CD4-Cre, St6gal1-flox/flox* (KO) mice and stained with SNA for flow cytometry analysis. n = 4-6.

(B – C) Peripheral blood samples were collected from young WT, young KO, or old WT mice and stained with ECL (B) or SLBR-N (C) for flow cytometry analysis. n = 7-9.

(D – H) Thymocytes from WT or KO mice were analysed using flow cytometry. An example of gating strategy is shown in (D). Total cellularity (E), early ($CD5^-$) and advanced ($CD5^+$) double positive (DP) T progenitors (F), formation of $Foxp3^+$ regulatory T cells (G), and immature ($TCRb^-$) or mature ($TCRb^+$) single positive (SP) $CD8^+$ T cells (H) were quantified respectively. n = 4-5.

All Data are represented in box plots showing the quartiles of the data with whiskers showing the rest of the distribution. *P* values were calculated by two-tailed student's t-test.

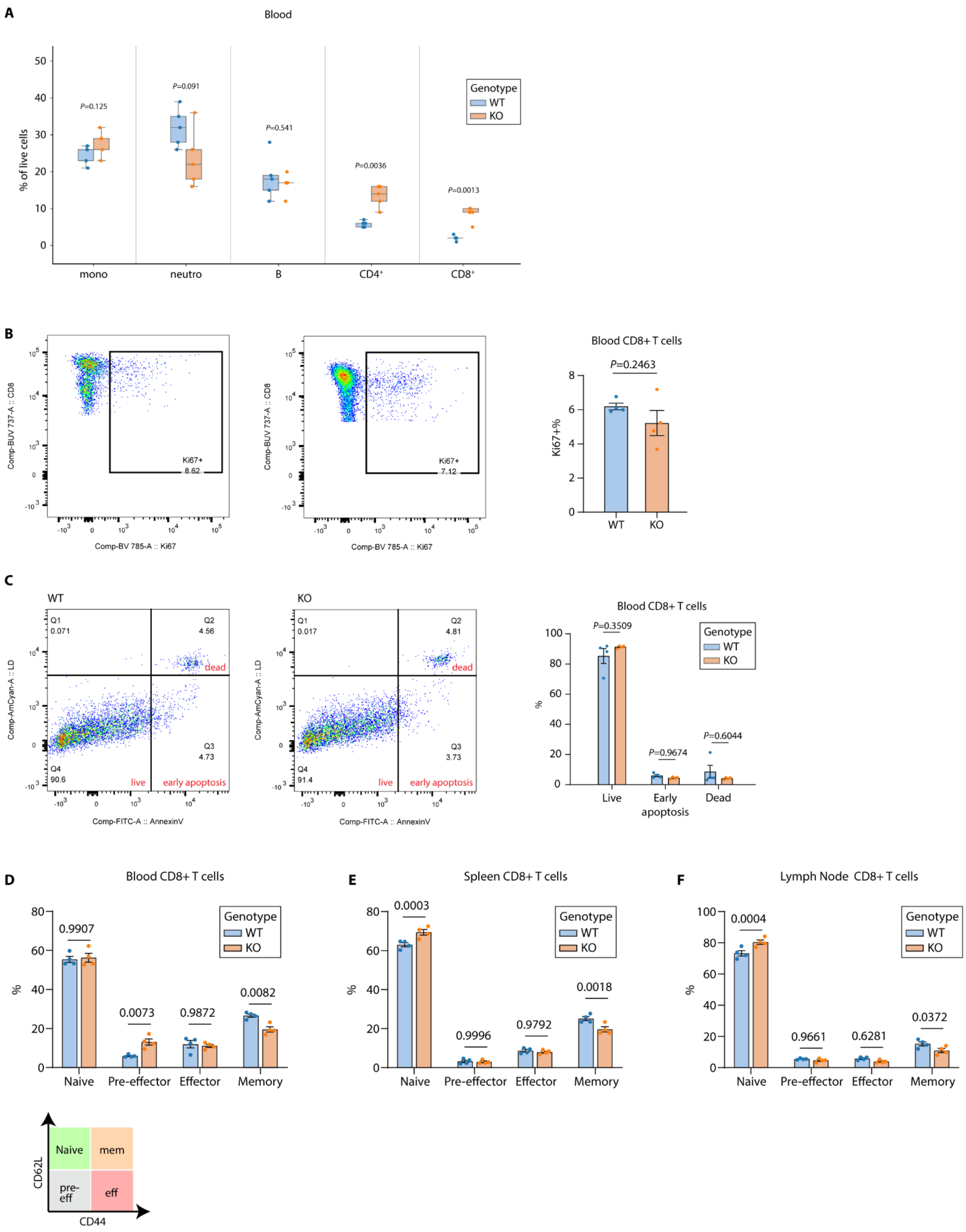

Figure S4. *St6gal1*-KO does not alter T cell proliferation or survival in the blood.

(A) Peripheral blood samples of WT and *St6gal1*-KO mice were analyzed using flow cytometry. n = 4-5.

(B) Peripheral blood samples of WT and *St6gal1*-KO mice were stained with Ki67 to evaluate proliferation of CD8+ T cells. n = 4.

(C) Peripheral blood samples of WT and *St6gal1*-KO mice were stained with LIVE/DEAD and Annexin V to evaluate CD8+ T cells proliferation. n = 4.

(D – F) CD8+ T cells from blood (A), spleen (B), and lymph node (C) were analyzed based on CD44 and CD62L staining to assess naïve, pre-effector (pre-eff), effector (effector memory, eff), and memory (central memory, mem) subpopulations. n = 4.

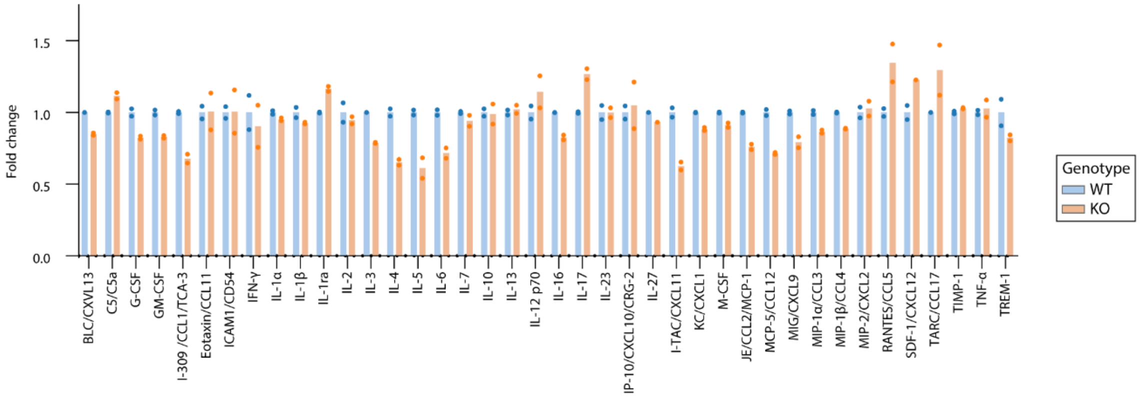

Figure S5. *St6gal1*-KO does not lead to increased overall inflammation

Serum samples from young WT or KO mice were collected for a cytokine array assay. n = 2 (technical replicates).

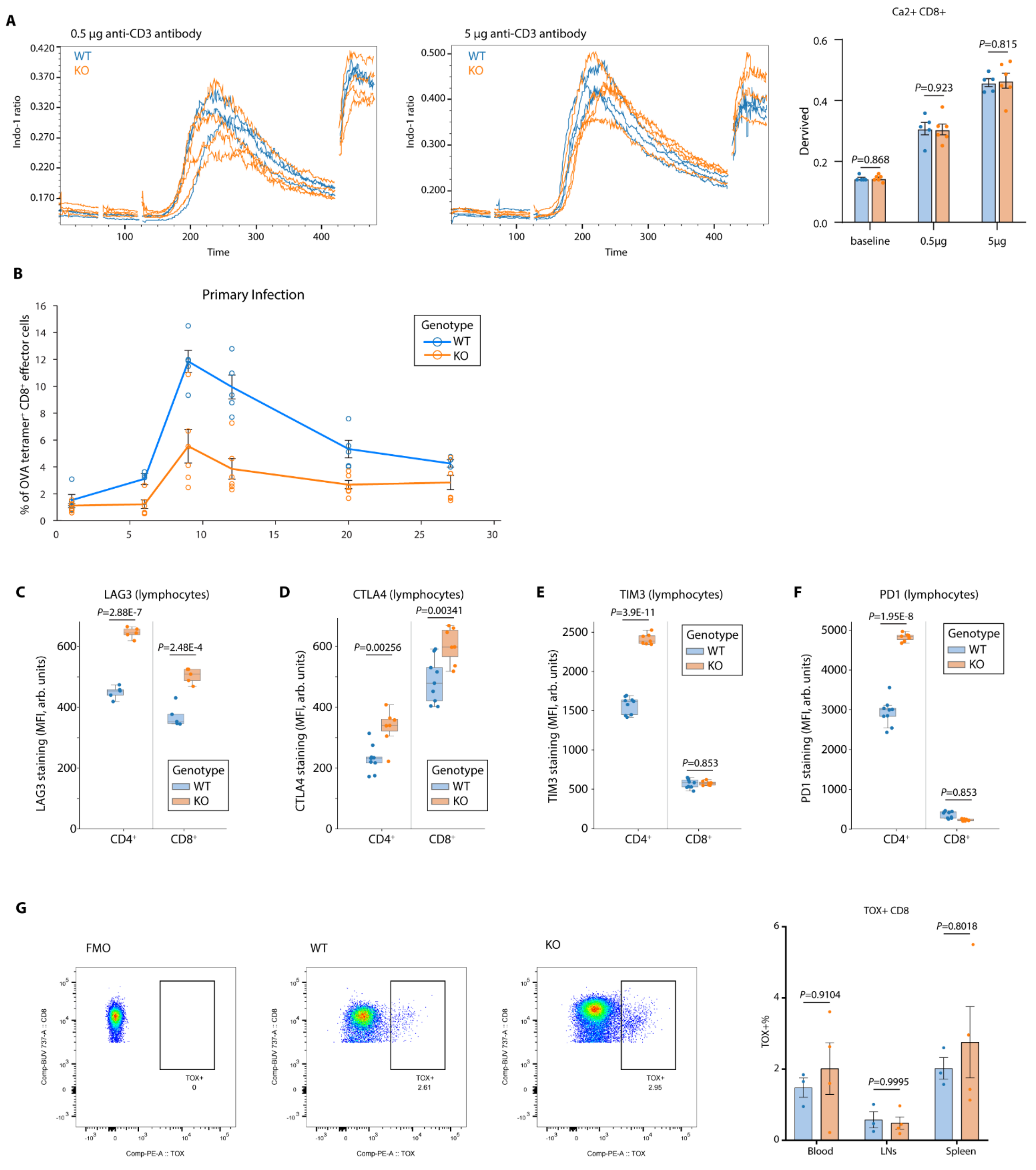

Figure S6. *St6gal1*-KO does not impact T cell activation sensitivity.

(A) Splenocytes from WT and KO mice were freshly prepared for Indo-1 calcium assay to evaluate CD8+ T cell activation sensitivity. n = 4.

(b) WT or KO mice were infected with Listeria-OVA on day 0 and blood samples collected on indicated days. OVA antigen-specific CD8+ T cells were analysed using flow cytometry. This panel is derived from Figure 4c and focuses on the primary response stage. n =5-6.

(C – F) T cell exhaustion markers including LAG-3 (C), CTLA-4 (D), TIM-3 (E), and PD-1 (F) were quantified on T cells in peripheral blood from wildtype or CD4-Cre, St6gal1$^{fl/fl}$ mice. n = 5-9. Data are represented in box plots showing the quartiles of the data with whiskers showing the rest of the distribution. *P* values were calculated by two-tailed student's t-test.

(G) Peripheral blood samples of WT and KO mice were stained with anti-TOX antibodies to evaluate T cell exhaustion status. n = 4.

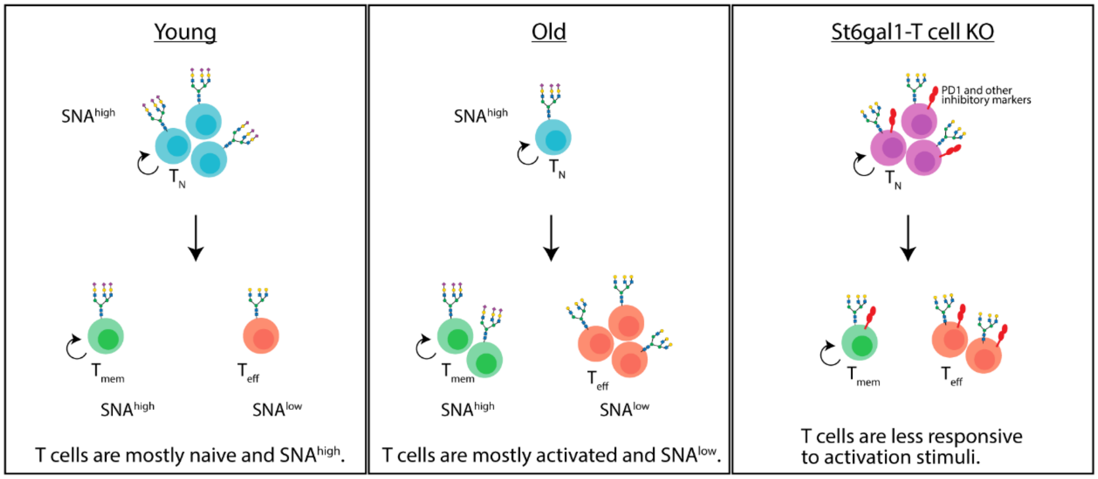

Figure S7. Proposed model of age-associated loss of α2,6-linked sialic acid and dampened T cell function.

A proposed working model of T cell aging-associated loss of α2,6-linked sialic acid. In young individuals, the majority of CD8+ T cells are naïve T cells with high levels of α2,6-linked sialic acid coating. In old individuals, the majority of CD8+ T cells become terminally differentiated effector T cells that are associated with depletion of α2,6-linked sialic acid. This loss of α2,6-linked sialic acid is not merely a consequence of T cell activation, but also directly affects T cell functions by upregulating inhibitory signaling.

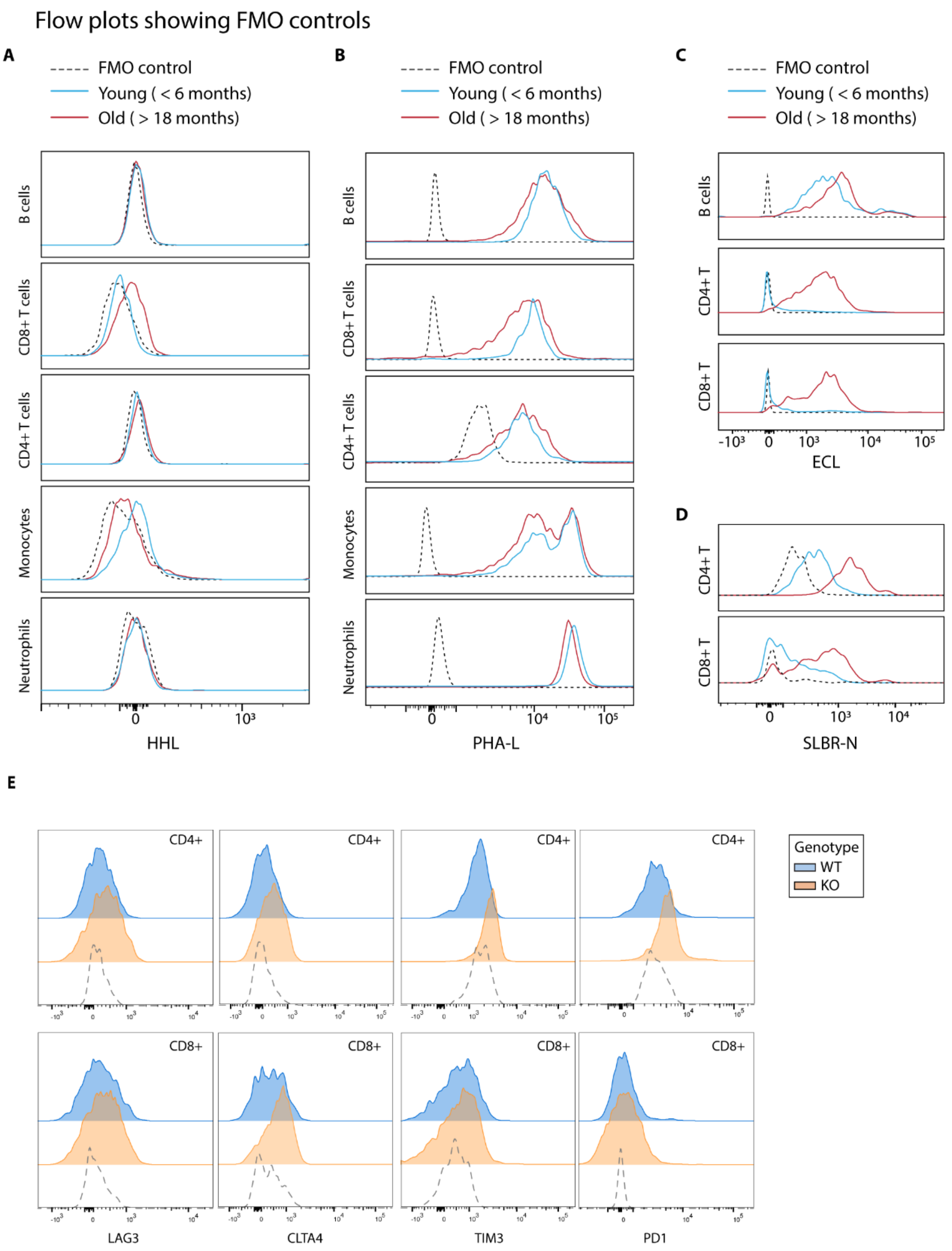

Figure S8. Flow data histograms showing FMO controls.

(A – D) Representative flow histograms of HHL (A), PHA-L (B), ECL (C), and SLBR-N (D) of peripheral blood samples from young and old mice.

(E) Representative flow histograms for LAG3, CLTA4, TIM3, and PD1 of CD4+ and CD8+ T cells in peripheral blood samples from WT and KO mice.

Supplementary Table 1: Lectin binding specificity

Lectin	Full name	predominant binding motif
HHL	Hippeastrum hybrid Lectin	5-8 N-glycan mannose structures
SNA	Sambucus nigra lectin	α2,6-linked sialic acid
SLBR-H	Siglec-like Binding Region-H	α2,3-linked sialic acid (sialyl-T antigen and sialyl Lewis C)
SLBR-N	Siglec-like Binding Region-N	α2,3-linked sialic acid (3’ sialyllactosamine and sialyl Lewis X)
ECL	Erythrina cristagalli lectin	LacNAc
PHA-L	Phaseolus vulgaris, leukoagglutinin	highly-branched N-glycans
